# Sustainable Working Life in a Swedish Twin Cohort—A Definition Paper with Sample Overview

**DOI:** 10.3390/ijerph18115817

**Published:** 2021-05-28

**Authors:** Annina Ropponen, Mo Wang, Jurgita Narusyte, Karri Silventoinen, Petri Böckerman, Pia Svedberg

**Affiliations:** 1 Division of Insurance Medicine, Department of Clinical Neuroscience, Karolinska Institutet, SE-171 77 Stockholm, Sweden; mo.wang@ki.se (M.W.); jurgita.narusyte@ki.se (J.N.); karri.silventoinen@helsinki.fi (K.S.); Pia.Svedberg@ki.se (P.S.); 2 Finnish Institute of Occupational Health, 00032 Työterveyslaitos, Finland; 3 Center of Epidemiology and Community Medicine, Stockholm County Council, 104 31 Stockholm, Sweden; 4 Population Research Unit, Faculty of Social Sciences, University of Helsinki, 00014 Helsinki, Finland; 5 School of Business and Economics, University of Jyväskylä, 40014 Jyväskylä, Finland; petri.bockerman@labour.fi; 6 Labour Institute for Economic Research, 00100 Helsinki, Finland; 7 IZA Institute of Labor Economics, 53113 Bonn, Germany

**Keywords:** systematic literature review, sustainable working life, labour market, prevalence, sick leave, unemployment

## Abstract

*Background*: A unified or consensus definition of “sustainable working life” remains lacking, although studies investigating risk factors for labour market exit are numerous. In this study, we aimed (1) to update the information and to explore a definition of “sustainable working life” via a systematic literature review and (2) to describe the working life trajectories via the prevalence of sickness absence (SA), disability pension (DP), and unemployment in a Swedish twin cohort to provide a sample overview in our Sustainable Working Life-project. *Methods*: A systematic literature review was conducted to explore the studies with the search phrase “sustainable working life” in PubMed, PsycInfo, and the Web of Science Database of Social Sciences in January 2021, resulting in a total of 51 references. A qualitative synthesis was performed for the definitions and the measures of “sustainable working life.” Based on the Swedish Twin project Of Disability pension and Sickness absence (STODS), the current dataset to address sustainable working life includes 108 280 twin individuals born between 1925 and 1990. Comprehensive register data until 2016 for unemployment, SA and DP were linked to all individuals. Using STODS, we analysed the annual prevalence of SA, DP, and unemployment as working life trajectories over time across education and age groups. *Results*: The reviewed 16 full articles described several distinct definitions for sustainable working life between 2007 and 2020 from various perspectives, i.e., considering workplaces or employees, the individual, organizational or enterprise level, and the society level. The definition of “sustainable working life” appearing most often was the swAge-model including a broad range of factors, e.g., health, physical/mental/psychosocial work environment, work motivation/satisfaction, and the family situation and leisure activities. Our dataset comprised of 81%–94% of individuals who did not meet SA, DP, or unemployment during the follow-up in 1994–2016, being indicative for “sustainable working life.” The annual prevalence across years had a decreasing trend of unemployment over time, whereas the prevalence of SA had more variation, with DP being rather stable. Both unemployment and DP had the highest prevalence among those with a lower level of education, whereas in SA, the differences in prevalence between education levels were minor. Unemployment was highest across the years in the youngest age group (18–27 years), the age group differences for SA were minor, and for DP, the oldest age group (58–65 years) had the highest prevalence. *Conclusions*: No consensus exists for a “sustainable working life,” hence meriting further studies, and we intend to contribute by utilising the STODS database for the Sustainable Working Life project. In the upcoming studies, the existing knowledge of available definitions and frameworks will be utilised. The dataset containing both register data and self-reports enables detailed follow-up for labour market participation for sustainable working life.

## 1. Introduction

A “sustainable working life” can be defined as the absence of disruptions and interruptions of working careers due to various reasons, including unemployment, rehabilitation, sickness absence (SA), and disability pension (DP) [1]. Furthermore, sustainable work refers to working and living conditions that support people in engaging and remaining in paid work throughout an extended working life [2]. Reducing the extent of work incapacity in terms of SA and DP is highly prioritised in the public policy of Nordic countries [3]. The SA and DP have increased in the industrialised world during the recent decades even when health conditions have generally improved at the same time, as seen, for example, in the increasing healthy life expectancy [4,5,6]. Another major concern is the overall inclusiveness of the working-age population in the labour market, which is emphasised by the Europe 2020 strategy for smart, sustainable, and inclusive economic growth [7].

The consequences of being absent from the labour market are severe; for example, SA/DP are linked to a number of negative health-related consequences, such as disease (the same disease for which SA/DP has been granted, or another disease), well-being, economy, career development, social integration, and premature death [8]. Even when substantial efforts have already been invested in investigating the risk factors for individuals’ absence from the labour market (i.e., SA/DP), there is still a gap in studies analysing factors that promote a “sustainable working life.” Furthermore, to the best of our knowledge, no consensus definition of “sustainable working life” exists, although many have approached this topic [1,2].

The Nordic countries provide excellent opportunities to investigate “sustainable working life” because their national registries are representative of their populations. Further, twin cohorts including comprehensive survey data with decades of follow-up have been collected in the Nordic countries, thus enabling the investigation of the role of both genetic factors and childhood environment in the association of predictive factors for “sustainable working life.” Using genetically informative data is important since the variation in complex phenotypes is caused by a combination of genetic and environmental factors and their mutual interactions [9]. The possibility to take into account genetic contributions to variation in “sustainable working life” is also important in order to show possible causality between various factors of interest and sustainable work-life participation. To the best of our knowledge, such studies have primarily focused on the associations between risk factors and interruptions, e.g., SA/DP or unemployment [10,11,12,13,14,15]. However, these earlier studies in which researchers of this article have been involved have shown that the heritability varies based on diagnosis groups for DP [16,17,18].

In this study, we utilised as the starting point the feasibility study of measuring “sustainable working life,” in which a systematic literature review was conducted for the period 2010–2017 [1]. This earlier review utilised “sustainable work” as a search term but found only a few relevant studies. Hence in order to identify more studies, the researchers included additional terms (i.e., NEET, work-life balance, and life course). Having this as the background, we designed a systematic literature review to update the information on “sustainable working life” and to explore a definition. Another aim was to describe the working life trajectories via the annual prevalence of SA, DP, and unemployment in a Swedish twin cohort to provide a sample overview in our Sustainable Working Life project. As an example of factors of interest for “sustainable working life,” we focused on SA, DP, and unemployment as they are the most common reasons for exit from the labour market.

## 2. Materials and Methods

### 2.1. Literature Review

**Figure 1:**
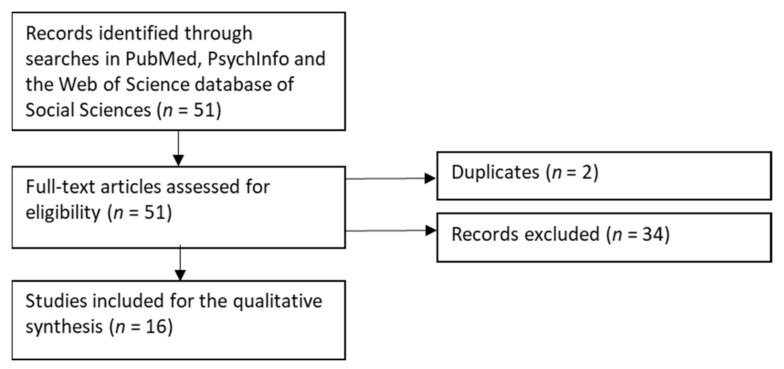
Flow diagram for systematic literature searches [20].

We conducted systematic literature searches in late January 2021 to explore the studies of “sustainable working life” published in English. Having the earlier review [1] as the starting point, we did not limit the time period of searches but utilised the search phrase “sustainable working life.” First, a literature search was conducted in PubMed (*n* = 36 references), PsycInfo (*n* = 2), and the Web of Science database of Social Sciences (*n* = 13) that resulted in 51 references in total (Figure 1). Then, all references were evaluated as full texts by 2 evaluators (AR and MW) who performed the evaluations separately and blinded [19]. The evaluation criterion applied was that the articles included a definition for “sustainable working life.” All types of articles and designs were considered. In 2 cases of discrepancy between evaluators, a third author (PS) made the tie-breaking decision regarding inclusion/exclusion. Due to wide variation in the designs, definitions, and measures of the included studies, we did not make formal comparisons or conduct a meta-analysis of these articles. Instead, we conducted a qualitative synthesis that provided the basis to collect the definitions of “sustainable working life” and the measures of “sustainable working life” utilised in the articles. After the removal of 2 duplicates, the main reasons for excluding the articles (*n* = 34) were no definition of “sustainable working life” or that the article was identified due to “department of sustainable working life” in one university (i.e., the search phrase existed in affiliations, not in the text of the article).

### 2.2. The Swedish Twin Project of Disability Pension and Sickness Absence

The Swedish Twin project Of Disability pension and Sickness absence (STODS) forms a national resource for genetic epidemiological studies regarding SA and DP but also for other labour market outcomes such as unemployment. STODS includes the twins identified in the Swedish Twin Registry (STR) who were born between 1925 and 1990, i.e., 119 907 twin individuals (approximately 1/3 are monozygotic [MZ], 1/3 same-sexed dizygotic [DZ], and 1/3 opposite-sexed DZ). Extensive survey data linked with the data obtained from national registers are already available. The survey data were collected through telephone interviews during the time period 1998–2002 (available for twins born 1925–1958) and through a Web-based questionnaire in 2005 (twins born 1959–1986) by STR. These data include background information (zygosity, age, and sex) and information on socioeconomic position (e.g., education), work-related factors (e.g., work history, work load, shift work, job insecurity, and Job demand-control-support (JDC-S) [21]), health (e.g., pain, musculoskeletal and mental disorders and common diseases), and health behaviour (e.g., physical activity, tobacco use, and alcohol consumption) [22,23]. Register data currently available for the time period 1994–2018 on DP (date, type, grade, and ICD diagnoses) and SA (grade and date of when each SA spell began and ended) and SA diagnoses for the time period 2005–2018 were collected from the Swedish Social Insurance Agency database MiDAS. Data on income, socioeconomic status, occupation, unemployment, old age pension, emigration, and rehabilitation currently available for the time period 1990–2016 were collected from Statistic Sweden (SCB) LISA database [24] and from other SCB databases that include corresponding information for other years not covered by LISA. The mortality data (date of death and diagnoses) were collected from the national death register, and data on non-fatal disease outcomes were collected from the inpatient and specialised outpatient registers held by the National Board of Health and Welfare. Register data for the STODS cohort are regularly updated.

As a data-based part for the sample overview of the data included in this Sustainable Work Life project, we described the annual prevalence (%) of the full sample without SA, DP, and unemployment for the time period 1994 to 2016 (*n* = 108 280, Supplemental Table S1), and we analysed the prevalence of SA, DP, and unemployment as working life trajectories until 2016. We also estimated differences in prevalence over time across education (measured as years of education and categorised as <10 years, 10–12 years, and >12 years) and age groups (categorised based on the distribution into 18–27 years, 28–37 years, 38–47 years, 48–57 years, and 58–65 years).

## 3. Results

### 3.1. Literature Review

The reviewed 16 full articles described several distinct definitions for “sustainable working life” (Table 1). Out of the articles, four were reviews, and six were qualitative studies complicating the evaluation of measures for “sustainable working life” (Table 2). The timeline of published studies indicated that “sustainable working life” is a relatively new concept since the earliest article with this specific phrase was published in 2007, and most were published in 2019 and 2020. Another aspect is region: six studies involving employees were conducted in Sweden as well as four of the reviews, whereas single studies were available from Australia, Italy, Netherlands, and the UK.

“Sustainable working life” seems to be defined from various perspectives, i.e., considering workplaces or employees, although some studies also suggested considering the individual level, the organizational and enterprise level, and the society level (Table 1). The definition of “sustainable working life” appearing most often was the swAge-model. This model defines “sustainable working life” to include health, physical work environment, mental/psychosocial work environment, working time and work pace, knowledge and competence, work motivation and work satisfaction, the attitude of managers and the organization/enterprise towards older workers, and the family situation and leisure activities (Table 1). In the swAge-model, the measures of “sustainable working life” would be:

1. Health effects of the work environment (and associations with biological age)
  - Function variation, diagnoses, and self-rated health
  - Physical working environment: Load, vibration, wear, dangerous substances, climate, access to tools, etc.
  - Mental work environment: Stress, demands, control, threats, violence, etc.
  - Working time, working rate, recovery: schedule, shifts, breaks, etc.
2. Finance (and associations with chronological age)
  - Economy: Personal financial situation, security, employability, insurance, etc.
3. Support and community (and associations with social age)
  - Private social environment: Private life, family life, and leisure in relation to work
  - Work social environment: Social support, discrimination, participation, attitudes, and leadership
4. Execution of task (and associations with cognitive age)
  - Work tasks, activity: Stimulation, motivation, and job satisfaction
  - Competence, knowledgeability, employability, and development in relation to the task

To sum up, the literature review indicated that definition and measures of “sustainable working life” vary to a large extent; therefore, no consensus on a definition exists.

### 3.2. Prevalence of Sickness Absence, Disability Pension and Unemployment as Working Life Trajectories

**Figure 2:**
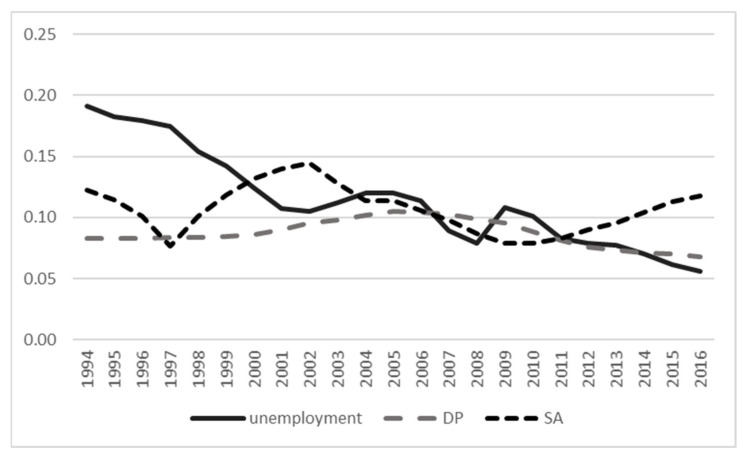
The annual prevalence of unemployment, disability pension (DP), and sickness absence (SA) from 1994 to 2016.

In our dataset of the full sample for those without unemployment, SA, or DP numbers varied between 82340 and 64189 from 1994 to 2016 and included 81%–94% of individuals who did not meet SA, DP, or unemployment during the follow-up (Supplemental Table S1). The annual prevalence across years is shown in Figure 2, indicating a decreasing trend of unemployment over time, whereas the prevalence of SA has had more variation and DP being rather stable.

**Figure 3:**
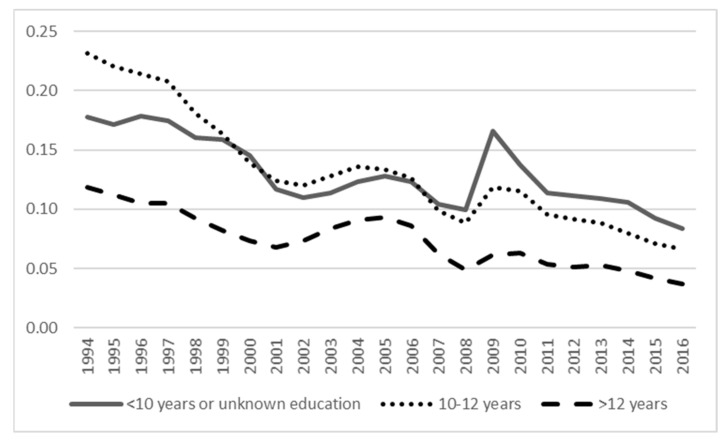
The prevalence of unemployment across categories of education.

**Figure 4:**
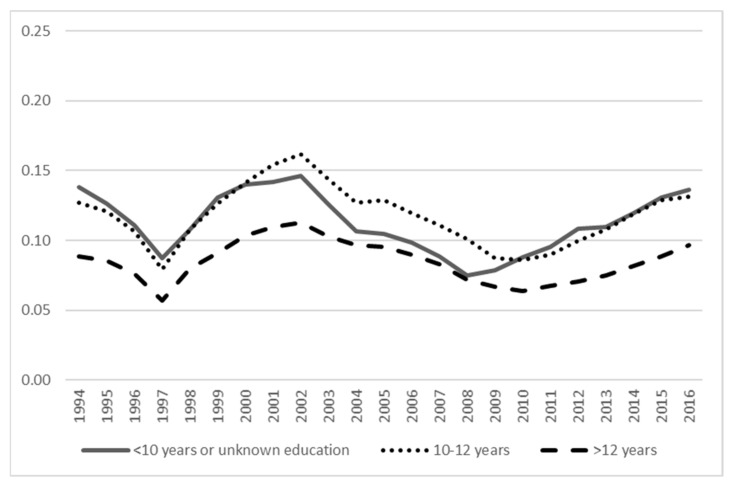
The prevalence of sickness absence across categories of education.

**Figure 5:**
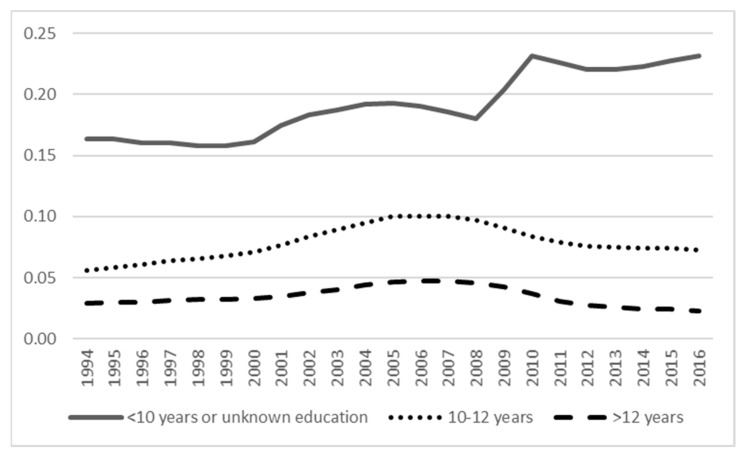
The prevalence of disability pension across categories of education.

Figure 3, Figure 4 and Figure 5 show the prevalence of unemployment, SA, and DP across education categories. Both unemployment and DP have the highest prevalence among those with a lower level of education compared to the highest level of education with the lowest prevalence. For SA, the differences in prevalence between education levels were minor. The Supplemental Figures S1–S3 show age group differences in the prevalence of unemployment, SA, and DP, indicating unemployment being highest across the years in the youngest age group (18–27 years), whereas for SA, the age group differences were minor (although the youngest age group had the lowest prevalence), and for DP, the oldest age group (58–65 years) had the highest prevalence.

## 4. Discussion

In this study, we conducted a systematic literature review to explore a definition and measures for “sustainable working life” and described the working life trajectories via the prevalence of SA, DP, and unemployment in a Swedish twin cohort as a cohort profile of our Sustainable Working Life project. In line with the feasibility study conducted in 2017 for measures of “sustainable working life” [1], the relevant studies were still few. In this study, we were not able to detect a unified definition of “sustainable working life,” although the swAge-model has gained interest in recent years [30,39]. The sample overview part of this study in which we estimated the annual prevalence of SA, DP, and unemployment, indicated variation across years for the follow-up from 1994 to 2016, but also that our sample included most (81%–94%) of individuals without such exit from the labour market. As an example of factors of interest for “sustainable working life” along with the swAge-model [39], we tested the effects of education and age on the prevalence of unemployment: SA and DP indicated differences across categories and even across time. Hence, our sample will have ample power and longitudinal design for further investigations of influential factors of “sustainable working life.”

### Strengths and Limitations

STODS is based on the population-based STR with comprehensive survey data collected by STR but with the addition of register data from several authorities covering the years 1994–2018, for almost 120,000 twin individuals. The twin data enable analyses controlling for familial confounding, which is genetics and mainly childhood and shared environment, hence extending the knowledge based on other samples without such possibility. Furthermore, the register data available for follow-up are detailed, including the date for starting and ending times of a spell for “sustainable working life” outcomes as well as diagnoses to define the analyses. Hence, we expect that we can contribute to the knowledge of “sustainable working life” through studies with the longitudinal design of genetically informative twins but also controlling and/or investigating many other influential factors for “sustainable working life” as identified in the systematic literature review part of this study. Even the measures suggested by the swAge-model [39] could be tested.

A limitation in the systematic review part of our study was that no consensus exists for the definition of “sustainable working life.” Although the topic has raised interest since 2010, many studies have created frameworks or lists of influential factors [1,37,39]. Another limitation is the regional emphasis: “sustainable working life” has raised particular interest in Sweden, limiting the generalisation and the applicability of the findings to Nordic countries only, where there are well-developed welfare systems, working life, and populations. The studies have also utilised various designs (i.e., from cross-sectional to longitudinal), methodologies (qualitative vs. quantitative), and samples (including studies with occupational groups, population-based samples or even students), whereas some studies were theoretical in nature. This further complicates the search for a definition or certain, commonly agreed on measures of “sustainable working life.” However, the expectation is that in our Sustainable Working Life project, we can add to the existing knowledge via the utilisation of available definitions and frameworks since our survey data cover self-reported aspects comprehensively. Furthermore, the register data enable detailed follow-up for labour market participation in terms of “sustainable working life,” as well as labour market non-participation in line with earlier studies based on STODS for SA, DP, and unemployment [10,41,42,43,44,45].

In conclusion, no consensus exists for the definition of “sustainable working life.” Hence, “sustainable working life” merits further studies, and we intend to contribute by utilising the STODS database for our Sustainable Working Life project.

## Figures and Tables

**Table 1:** Comparative analysis of the definitions for “sustainable working life” in terms of target population and theoretical model or related measure.

Year	Author(s) (Ref)	Definition	Comparative Features	Number of Citation	Time-Span of Citations
2007	Kaneklin and Gorli [25]	Sustainable working life is the capacity for organizations to create and regenerate value through the application of participative policies and practices to promote both organizational performances and people’s well-being. Relief of social dimension that inhabits organizational changes and maintain it close to the functional and strategic organizational changes, since the structural and functional dimensions of an organization must come together with the social and cultural dimensions for a sustainable working life.	Population: Healthcare sector organizations in Italy. Design: Action research.	2	2007–2020
2013	Hansen et al. [26]	Personal- and practice-based, professional, and systemic themes containing a number of sub-themes representing the experiences (i.e., ability to work part-time, achieve a healthy work-life balance, etc.), initiatives (e.g., alternatives to ownership of practice or develop teams with multidisciplinary support), or conditions (e.g., payment systems supportive of continued involvement in teaching or educational opportunities) that promote a long and sustainable working life in rural general practice.	Population: Australian rural general practitioners >45 years on age. Design: Semi-structured qualitative interview.	0	-
2013	Koolhaas et al. [27]	The increase in problems due to ageing and health-related problems from the age of 45 years onwards implies the importance of attention to obstacles and retention factors for maintaining or enhancing a sustainable working life.	Population: Workers aged ≥45 years in nine different companies in Netherlands. Design: A cross-sectional in-depth survey.	13	2013–2020
2015	Leider et al. [28]	Sustainable working life consists of parameters: work ability, productivity, vitality, and/or work role functioning.	Population: - Design: Systematic literature review utilizing Web of Science, Medline/PubMed and Embase to identify papers published in peer-reviewed journals between 1997 and 2013.	42	2015–2020
2015	Vänje [29]	Sustainable working life includes the perspectives of crafting employees’ individual resources as well as collaboration between employees and their managers in order to create organizational development.	Population: - Design: Literature review using the Royal Institute of Technology’s (KTH’s) library search engine KTHB Primo, EndNote and the Social Sciences Citation Index at Web of Science (ISI), the Swedish search engines LIBRIS (http://libris.kb.se/) and KVINNSAM (http://www.ub.gu.se/kvinn/kvinnsam/) from the mid-1980s until 2014.	5	2015–2019
2016	Nilsson [30]	Four different concepts of ageing; the nine factors of importance for working life; and their relation to older workers’ decision to extend their working life or retire. Employees’ biological ageing is important due to individual health and well-being in association with their work situation (work pace, time, and environment); employees’ chronological ageing involves statutory retirement age, social insurance, policies and economic incentives in working life and society. Adequate personal finances, i.e., providing for a living, food, and essential factors, but also motivation factors (e.g., the possibility for social inclusion/participation in an inspiring work situation and for motivating and stimulating activities and tasks based on the individuals’ knowledge) are important.	Population: - Design: Literature review in Medline, PubMed, Scopus, Science Direct, Web of Knowledge, Cochrane Library and Google Scholar, in addition Swedish library database LIBrIS and Lund University library database, and also the Organisation for economic Co-operation and Development (OECD), the World Health Organisation (WHO), the World economic Forum (WeF), and the European Union (EU) in 2003–2015.	7	2016–2020
2017	Eriksson et al. [31]	Sustainable workplaces as work environments that embrace factors that contribute to employee health and well-being, as well as organizational efficiency. By integrating human and economic values, sustainable workplaces can even impact societal effectiveness.	Population: - Design: Scoping review in Web of Science, Scopus, Pubmed, Cinahl, Academic Search Premier, PsycInfo, and Embase for 2009–2014.	3	2019–2020
2018	Forslin et al. [32]	A well-functioning balance between a working and private life is important for a sustainable working life over time.	Population: Those with a definite MS diagnosis and an outpatient appointment with a neurologist in Sweden and alive, of working age at the 10-year follow-up (<55 years of age at baseline). Design: A 10-year longitudinal observational study.	5	2019–2020
2018	Wålinder et al. [33]	Social support and low strain (JCD-model) are linked with workers’ well-being and a sustainable working life in the health-care sector.	Population: Hospital workers in university hospitals in Sweden Design: Cross-sectional survey study	0	-
2019	Gyllensten et al. [34]	Sustainable working life according to swAge-model depends on health, physical work environment, mental/psycho-social work environment, working time and work pace, knowledge and competence, work motivation and work satisfaction, the attitude of managers and the organisation/enterprise towards older workers, the family situation, and leisure activities.	Population: Employees of health and elderly care homes in Sweden Design: Focus group interviews	1	2019
2019	Thompson et al. [35]	The concept of sustainable working life includes organizations devising career paths that support staff to retain their health (physical and mental), productivity, and motivation over an extended period of employment. Vulnerable employees may cycle between the more- and the less-adaptive poles of each chronotope, and even between chrontopes, given that people with chronic illness are known to draw on a range of self-management strategies over time.	Population: Multiplesclerosis patients in UK. Design: Dialogical analysis of focus group interviews.	0	-
2020	Blomé et al. [36]	The swAge-model: the individual motives and considerations for continuing to work and the older workers’ retirement decisions are based on: (a) their health in relation to the work situation and work environment versus health in retirement; (b) their personal economic situation in employment versus in retirement; (c) their opportunities for social inclusion in working life situations versus in retirement; (d) their opportunities for meaningful and self-crediting activities in working life versus in retirement	Population: Focus group interviews of 3–7 older workers, managers, trade union representatives, and human resource personnel from public organizations, large private companies and from private small-to-medium-sized enterprises in Sweden. Design: Secondary analysis of age management with the theoretical swAge- model.	3	2020
2020	Gyllensten et al. [37]	Continuing to work at an older age is determined by “push factors,” i.e., chronic diseases, physical demands, and poor working conditions, and “‘pull factors,” such as one’s spouse not working, care-taking of relatives, and leisure time expectations. Additionally, norms about working and retiring, economic incentives, attitudes at the workplace, work satisfaction, and social relationships at work and home are important factors for extended working life.	Population: All individuals employed at one car manufacturer in Sweden during 2005–2015. Design: A case-control study for 10-year follow-up.	0	-
2020	Lindmark et al. [38]	Focus on the prevention of ill health, health-promoting factors (e.g., occupational balance, emotional intelligence, social interaction/teamwork) for improvement of people’s capacity to develop abilities and resources to feel good and cope with different situations in a healthy way are essential for health and sustainable working life.	Population: Students of higher education programs in the healthcare and social work sectors in Sweden. Design: Baseline results of a multicentre longitudinal study.	0	-
2020	Nilsson [39]	The swAge-model describes three influence levels of importance for work-life participation and to a sustainable, extended working life: the individual level, micro level; the organizational and enterprise level, meso level; and the society level, macro level.	Population: - Design: Descriptive for swAge-model which will be developed based on grounded theory using qualitative and quantitative studies, intervention projects, and literature reviews.	1	2020
2020	Nunstedt [40]	A reduced workload, varied tasks, individual schedules, clear leadership, and cooperation between nurses and other professionals are factors that contribute to a good working climate, sense of coherence, and meaningfulness. Hence, these can be used for action programmes, which, in turn, can promote a sustainable working life.	Population: Nurses in a hospital in western Sweden. Design: Qualitative and descriptive in design including a literature review, interviews, a qualitative content analysis, and a deductive approach for theoretical discussion.	1	2021

**Table 2:** Suggested measures for “sustainable working life” in terms of source population or data.

Author(s) (Ref)	Measure	Source
Kaneklin and Gorli [25]	n.a.	*n* = 14 middle managers
Hansen, Pit, Honeyman, and Barclay [26]	Encouragement and support at all stages of career, wishing to work part-time. Try to achieve control over your working life by maintaining a healthy work-life balance through the implementation of mental and lifestyle strategies. Eat healthily, be physically active, and recognise and respond to signs of stress and burnout. Support and assistance to those wishing to sell their practice but remain at work Work in a good team and promote good team communication through regularly scheduled meetings. Have a gradual retirement plan. Promotion of practice structures enabling to retire gradually without being financially penalised. Implement practice-based health promotion strategies. Pursue a special professional interest. Become involved in teaching and mentoring young workers. Implement legislation to make it financially viable for semi-retires to remain at work Ensure that a good range of educational opportunities are available and easily accessible Reduce the bureaucratic burden Implement strategies to improve the status and recognition. Build on and improve utilisation of the current local locum database.	*n* = 16
Koolhaas, van der Klink, Vervoort, de Boer, Brouwer, and Groothoff [27]	Workers’ perspectives on problems, obstacles, retention factors, and needs due to ageing classified with the International Classification of Functioning, Disability and Health (ICF).	*n* = 3008 workers, response rate 36%
Leider, Boschman, Frings-Dresen, and van der Molen [28]	Job rotation comprises rotating between tasks within jobs and/or between activities Exposures related to musculoskeletal complaints Sustainable working life: work ability, productivity, vitality, and/or work role functioning.	Search terms: job rotation, musculoskeletal complaints, terms for related exposures and terms for sustainable working life parameters. *n* = 16 included studies.
Vänje [29]	n.a.	
Nilsson [30]	Health; economic incentives; family, leisure, and surrounding society; physical work environment; mental work environment; work pace and working hours; competence and skills; motivation and work satisfaction; and the attitude of managers and organisation to older workers.	Discourse analysis of documents was used in an integrative review including 128 articles.
Eriksson, Orvik, Strandmark, Nordsteien, and Torp [31]	n.a.	In-depth analysis of 20 studies
Forslin, Fink, Hammar, von Koch, and Johansson [32]	Employment status at the 10-year follow-up categorised as full-time work, part-time work (working, but less than full time), and no work.	Baseline and follow-up surveys, *n* = 154
Wålinder, Runeson-Broberg, Arakelian, Nordqvist, Runeson, and Rask-Andersen [33]	Well-being at work Zest for work (i.e., emotions about work) Intention to stop working with health care	*n* = 1405 hospital employees
Gyllensten, Wentz, Håkansson, Hagberg, and Nilsson [34]	Organisational issues High demands Lack of staff Lack of recovery at work Health-related problems Tiredness and aches Individually created solutions to cope with chronic health problems Private issues Poor personal finances postpone retirement Lack of private life Meaningfulness and appreciation Meaningful job Downgrading of competencies Social support Belonging Support from colleagues increases motivation for delaying pension	*n* = six focus groups with four to eight participants in each group
Thompson, Ford, Stroud, and Madill [35]	n.a.	Dialogical analysis of 20 workers
Blomé, Borell, Håkansson, and Nilsson [36]	Contemporary policies and practice in the work environment Social participation and attitudes Experience and mentorship	Qualitative interviews, *n* = 16
Gyllensten, Torén, Hagberg, and Söderberg [37]	Employers’ register for employment status: active at work, retired (either retired at the age 55–62 or working ≥63 years during the observation years)	*n* = 572 cases and 771 controls
Lindmark, Ahlstrand, Ekman, Berg, Hedén, Källstrand, Larsson, Nunstedt, Oxelmark, Pennbrant, Sundler, and Larsson [38]	Health-promoting dimensions: Health-promoting resources (i.e., sense of coherence) Occupational balance Emotional intelligence Health and welfare Social interaction Work and workplace experiences/perception	*n* = 2283 students
Nilsson [39]	1. Health effects of the work environment (and associations with biological age) Function variation, diagnoses, and self-rated health Physical working environment: Load, vibration, wear, dangerous substances, climate, access to tools, etc. Mental work environment: Stress, demands, control, threats, violence, etc. Working time, working rate, recovery: schedule, shifts, breaks, etc. 2. Finance (and associations with chronological age) Economy: Personal financial situation, security, employability, insurance, etc. 3. Support and Community (and associations with social age) Private social environment: Private life, family life, and leisure in relation to work Work social environment: Social support, discrimination, participation, attitudes, and leadership 4. Execution of task (and associations with cognitive age) Work tasks, activity stimulation, motivation, and job satisfaction Competence, knowledgeability, employability, and development in relation to the task	Grounded theory
Nunstedt [40]	Job satisfaction Professional role Job engagement Belonging in the workplace Working conditions and factors for remaining in the profession Opportunities for learning and development in the workplace The professional role in the future	*n* = 12

n.a. = not applicable.

## Data Availability

The data presented in this study are not publicly available. Readers may contact the last author regarding details. The data are not publicly available due to the legal restrictions set out in the General Data Protection Regulation, the Swedish law SFS 2018:218, the Swedish Data Protection Act, the Swedish Ethical Review Act, and the Public Access to Information and Secrecy Act. These types of sensitive data can only be made available after legal review, for researchers who meet the criteria for access to these types of sensitive and confidential data.

## Supplementary Materials

The following are available online at https://www.mdpi.com/article/10.3390/ijerph18115817/s1, Table S1. The full sample, prevalence of those without unemployment, sickness absence or disability pension pre a year from 1994 to 2016, Figure S1. Prevalence of unemployment across age categories, Figure S2. Prevalence of sickness absence across age categories, Figure S3. Prevalence of disability pension across age categories.
