# Supplementary material for: Sustainable Working Life in a Swedish Twin Cohort—A Definition Paper with Sample Overview
Source: Int J Environ Res Public Health. 2021 May 28;18(11):5817. doi: 10.3390/ijerph18115817 (PMC8197988; doi:10.3390/ijerph18115817)
Supplement: Supplementary file 1 [file ijerph-18-05817-s001.zip › ijerph-1201535-supplementary.pdf]

International Journal of
Environmental Research and Public Health

MDPI

Supplemental material

Table S1. The full sample, prevalences of those without unemployment, sickness absence or disability pension pre a year from 1994 to 2016.

	No unemployment			No sickness absence			No disability pension		
year	Full sample	Frequency	%	Full sample	Frequency	%	Full sample	Frequency	%
1994	81488	65910	81	81682	71683	88	81682	74884	92
1995	81344	66516	82	81555	72219	89	81555	74765	92
1996	81293	66705	82	81511	73263	90	81511	74751	92
1997	81357	67150	83	81565	75331	92	81565	74736	92
1998	81337	68779	85	81519	73269	90	81519	74722	92
1999	81342	69751	86	81533	71846	88	81533	74673	92
2000	81375	71255	88	81560	70823	87	81560	74554	91
2001	81388	72616	89	81575	70181	86	81575	74263	91
2002	81654	73054	89	81877	70006	86	81877	74083	90
2003	81854	72664	89	82070	71535	87	82070	74003	90
2004	81702	71873	88	81927	72613	89	81927	73550	90
2005	81688	71849	88	81871	72551	89	81871	73294	90
2006	81861	72546	89	82057	73351	89	82057	73533	90
2007	82049	74745	91	82244	74204	90	82244	73825	90
2008	82149	75688	92	82340	75207	91	82340	74244	90
2009	79735	71090	89	79907	73579	92	79907	72303	90
2010	77329	69508	90	77496	71386	92	77496	70616	91
2011	74957	68739	92	75116	68915	92	75116	69031	92
2012	72650	66914	92	72819	66264	91	72819	67304	92
2013	70457	65028	92	70599	63878	90	70599	65439	93
2014	68157	63380	93	68290	61166	90	68290	63413	93
2015	66103	62046	94	66238	58774	89	66238	61578	93
2016	64046	60479	94	64189	56657	88	64189	59813	93

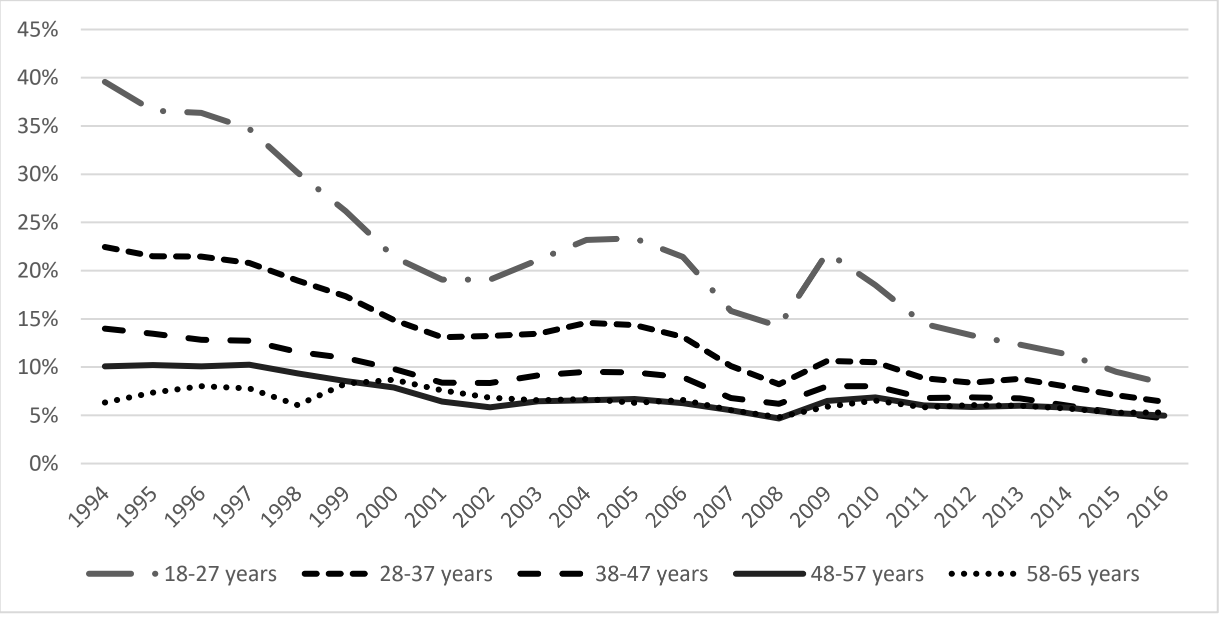

Figure S1. Prevalence of unemployment across age categories.

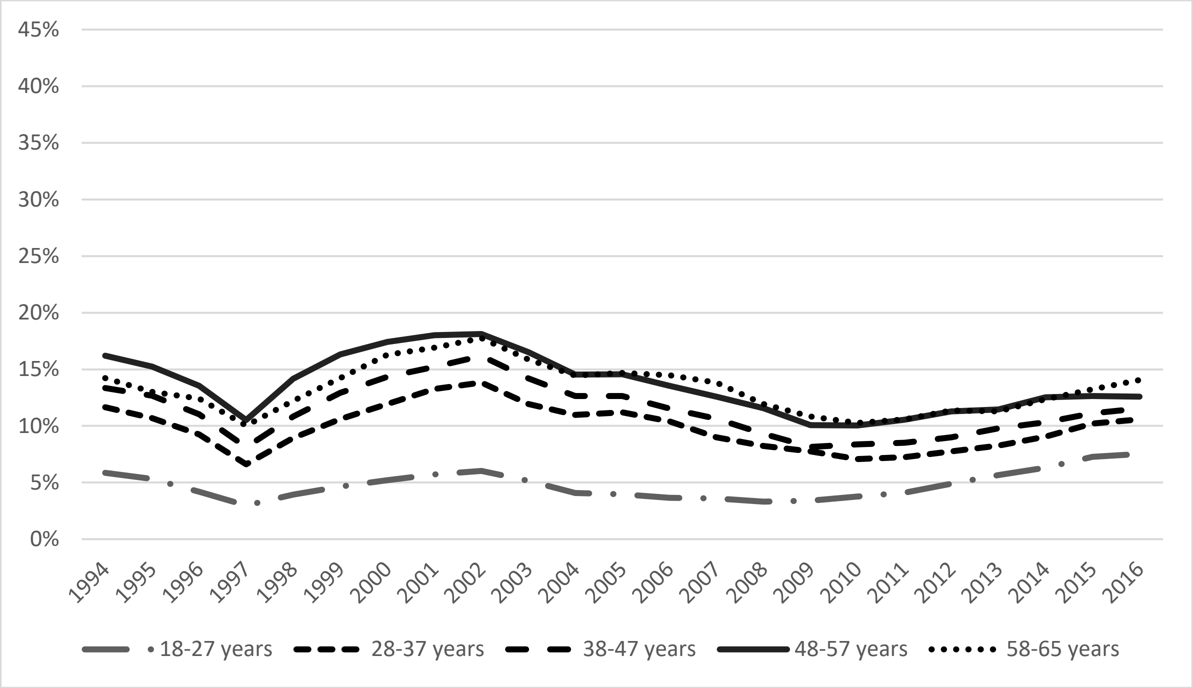

Figure S2. Prevalence of sickness absence across age categories.

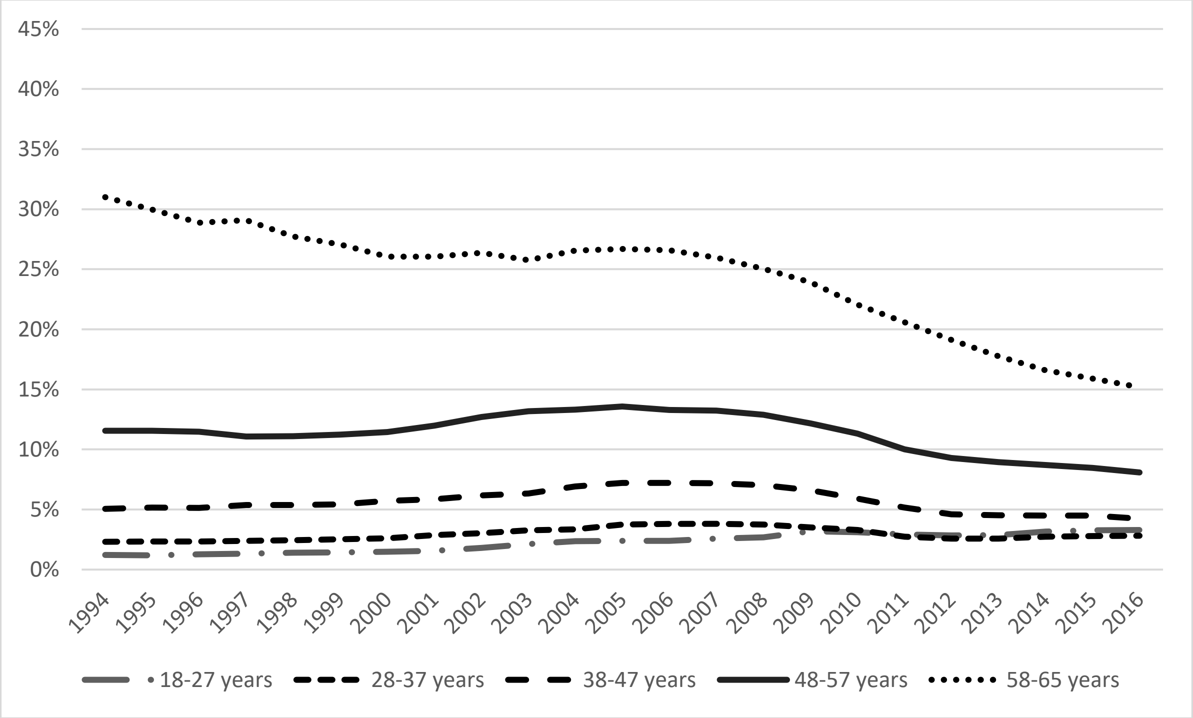

Figure S3. Prevalence of disability pension across age categories.
